# New Insights Into the Pathogenesis of Bullous Pemphigoid: 2019 Update

**DOI:** 10.3389/fimmu.2019.01506

**Published:** 2019-07-02

**Authors:** Giovanni Genovese, Giovanni Di Zenzo, Emanuele Cozzani, Emilio Berti, Massimo Cugno, Angelo Valerio Marzano

**Affiliations:** ^1^Dermatology Unit, Fondazione IRCCS Ca' Granda Ospedale Maggiore Policlinico, Milan, Italy; ^2^Department of Physiopathology and Transplantation, Università degli Studi di Milano, Milan, Italy; ^3^Molecular and Cell Biology Laboratory, Istituto Dermopatico dell'Immacolata (IDI) IRCCS, Rome, Italy; ^4^DISSAL Section of Dermatology, Università degli Studi di Genova, Genoa, Italy; ^5^Internal Medicine Unit, Fondazione IRCCS Ca' Granda Ospedale Maggiore Policlinico, Milan, Italy

**Keywords:** bullous pemphigoid, pathogenesis, autoantibodies, autoimmunity, skin, autoimmune blistering diseases

## Abstract

There are several lines of evidence indicating that the physiopathological bases of bullous pemphigoid (BP), the most common subepidermal autoimmune bullous disease, are hallmarked by the production of autoantibodies directed against the hemidesmosomal anchoring proteins BP180 and BP230. In contrast to the robustness of the latter assumption, the multifaceted complexity of upstream and downstream mechanisms implied in the pathogenesis of BP remains an area of intense speculation. So far, an imbalance between T regulatory cells and autoreactive T helper (Th) cells has been regarded as the main pathogenic factor triggering the autoimmune response in BP patients. However, the contributory role of signaling pathways fostering the B cell stimulation, such as Toll-like receptor activation, as well as that of ancillary inflammatory mechanisms responsible for blister formation, such as Th17 axis stimulation and the activation of the coagulation cascade, are still a matter of debate. In the same way, the pathomechanisms implied in the loss of dermal-epidermal adhesion secondary to autoantibodies binding are not fully understood. Herein, we review in detail the current concepts and controversies on the complex pathogenesis of BP, shedding light on the most recent theories emerging from the literature.

## Introduction

Bullous pemphigoid (BP), the most common autoimmune bullous disease, typically presents with generalized crops of tense, pruritic cutaneous blisters and mostly affects the elderly (1). In up to 20% of cases, BP may initially exhibit a non-bullous phase characterized by eczematous, excoriated, urticaria-like or nodular lesions, which may last weeks, months, or occasionally remain the sole clinical manifestation (2). Together with mucous membrane pemphigoid, pemphigoid gestationis, linear immunoglobulin (Ig) A bullous dermatosis (LABD), anti-laminin γ1 pemphigoid, and epidermolysis bullosa acquisita (EBA), BP is encompassed in the heterogeneous group of subepithelial autoimmune bullous disorders (1). Indeed, the histopathological assessment of a recent blister generally reveals a dermal-epidermal split associated with a dermal inflammatory infiltrate mainly consisting of lymphocytes and eosinophils (3). In BP, blisters are caused by autoantibodies of the IgG class directed against two structural components of the hemidesmosome, a multiprotein complex of the dermal-epidermal junction providing structural adhesion between basal keratinocytes and dermal extracellular matrix; these antigens are BP180, a transmembrane glycoprotein consisting of a globular cytoplasmic N-terminal domain, a short transmembrane stretch and a large extracellular C-terminal domain containing 15 collagenous repeats (4–7), and BP230, a protein of the hemidesmosomal inner plaque with a central rod domain flanked by globular end domains (4, 5). In addition to IgG also IgE autoantibodies are involved in disease pathogenesis and could be detected in the skin and/or serum of BP patients by means of immunofluorescence studies, immunoblot/immunoprecipitation and enzyme-linked immunoassay (ELISA) analyses (8). Whilst the crucial role of these autoantibodies in triggering the inflammatory cascade is fully acknowledged (9), many ancillary mechanisms involved in the complex etiology of BP need to be elucidated yet (10). In this regard, the development of animal models, which include the transfer of autoantibodies to experimental animals, the adoptive transfer of autoantigen-specific B lymphocytes to immunodeficient mice and immunization-induced models, greatly contributed to dissect these pathogenic aspects (11). This review provides a breakdown of the most recent studies on the pathogenic mechanisms involved in BP.

## Established Status of Knowledge on Bullous Pemphigoid Pathogenesis

**Figure 1:**
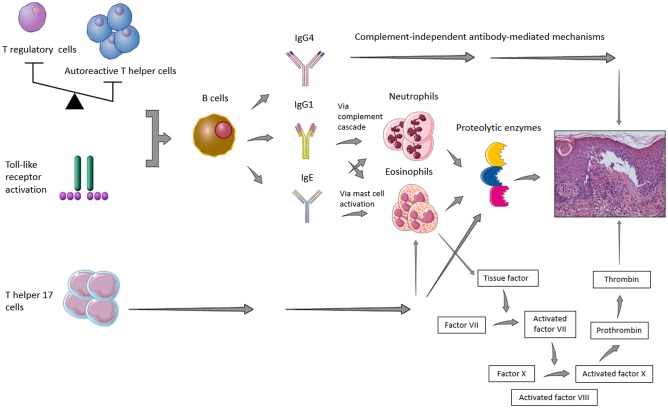
Representative figure of the main pathogenic pathways implied in blister formation in patients with bullous pemphigoid.

It has been recognized that a combination of genetic predisposing factors, such as class II HLA (e.g., HLA-DQβ1^*^0301) (12), and environmental influences, such as UV radiation, traumas, and drugs, may contribute to the loss of immune tolerance toward the above-mentioned antigens of the dermal-epidermal junction (13). As shown in Figure 1, it has also been suggested that the imbalance between autoreactive T helper (Th) and T regulatory (T_reg_) cells (14, 15) as well as a T cell-independent activation of toll-like receptor (TLR) system (16) may induce B cell stimulation, with consequent BP autoantibody secretion. In parallel, the Th17 pathway activation appeared to maintain the inflammatory cascade started by humoral hyperactivation, by triggering Th2 response, recruiting neutrophils and eosinophils and stimulating the release of proinflammatory cytokines and proteolytic enzymes (14, 17).

While the role of IgA or IgM is still elusive and their deposits did not seem to affect the disease course (18), the role of IgE is controversial. Some authors demonstrated that their deposits along the basement membrane zone (BMZ) or their serum levels correlated to specific BP clinical features, namely more infiltrated lesions, such as urticarial plaques (18–20) or nodules (21), while other ones observed no association between circulating anti-BP180 IgE antibodies and a non-bullous urticaria-like presentation (21, 22). Furthermore, serum anti-BP180 IgE levels were shown to reflect disease activity throughout the course of the disease (21, 22), even if it has been suggested that it might not happen in the early phases of medication (23). However, on direct immunofluorescence studies IgG are usually predominant. Mihai et al. (24) showed that IgG1 and IgG4 were the most frequent IgG subtypes found in BP along the dermal-epidermal junction. By means of a cryosection assay, the same authors proved also that IgG1 promoted blister formation through complement fixation, whereas IgG4, which cannot fix the complement, activated leucocytes and induced dermal-epidermal separation with a lower pathogenic potential as compared to IgG1 (24).

BP180 is the main BP autoantigen and IgG and/or IgE against this antigen may be detected in most patients (8). The immunodominant non-collagenous region 16A (NC16A) of the BP180 ectodomain is extensively accepted to be the main target for BP autoantibodies. However, also domains of BP180 outside of NC16A, such as the C-terminal domain of BP180 and a 120-kDa fragment of BP180 known as LAD-1, may be recognized (25–27). The IgG directed against BP180 domains other than NC16A seemed to be responsible of less inflammatory manifestations (28) due to the fact that they hardly induce any BP180 depletion respect to anti-BP180-NC16A IgG in experimental studies (29). Moreover, IgG reactivity with extracellular but not intracellular epitopes of BP180 paralleled the severity of the disease course (25) and higher levels of anti-BP180 IgG were associated with increased 1-year mortality (30). The pathogenic role of IgG anti-BP180 has been validated by the following remarks: (i) the induction of BP-like lesions in mice in which anti-BP180-NC16A antibodies had been passively transferred (31); (ii) the onset of BP-like bullae and a human disease phenotype in BP180-knockout mice rescued by the human ortholog after being injected with IgG from BP patients (32); (iii) the direct correlation of anti-BP180-NC16A antibody titers to disease activity (33, 34). Anti-BP180 IgE antibodies showed also pathogenic features; indeed, the pathogenicity of anti-BP180 IgE antibodies has been demonstrated via an IgE hybridoma to LABD97, a component of the shed ectodomain of BP180, which was injected in severe combined immunodeficiency (SCID) mice with engrafted human skin, with consequent development in the skin grafts of eosinophil infiltration and histological subepidermal blisters resembling those of BP (35). Furthermore, the injection of total IgE isolated from two BP patients sera into human skin grafted onto athymic nude mice induced erythematous plaques reminiscent of those clinically seen in BP (36). Antibodies of the IgG class reacting against BP230, combined or not with anti-BP180 antibodies, are usually found in a smaller proportion of BP patients (37). Although the actual pathogenic role of anti-BP230 autoantibodies has been initially debated (38, 39), it has been more recently well-documented (40, 41) and IgG reactivity with intracellular epitopes of BP230 has been shown to correlate to BP severity (25).

The pathogenic mechanisms cooperating in the blister formation secondary to BP autoantibody binding to their targets are complex and may be subdivided into complement-dependent and complement-independent ones. Indeed, IgG1 have been demonstrated to start the inflammatory complement cascade leading to the recruitment of neutrophils and eosinophils in BP, and, consequently, to release of proteolytic enzymes in an experimental mouse model of BP (42). In addition to complement-mediated mechanisms, more and more evidence emerges that also complement-independent pathways play a fundamental role in BP pathogenesis. It has been postulated that there may be a direct influence of autoantibodies in adhesion functions of autoantigens. Indeed, Iwata et al. (43) disclosed that serum IgG from BP patients could deplete cultured keratinocytes of BP180 and decrease cell adhesion to the bottom of the culture plate, thus suggesting that hemidesmosomes had an insufficient adhesive function due to a reduced hemidesmosomal BP180 content. Furthermore, Ujiie et al. showed that passive transfer of BP autoantibodies caused BP180 depletion in lesional skin independently by complement deposition in neonatal C3-deficient BP180-humanized mice (44). The role of IgG4 in inducing the blister formation without complement cascade intervention has been supported by Dainichi et al., who reported two cases of IgG4-positive BP without complement activation (45). Mast cells also have been proposed to contribute to BP development and the presence of IgE autoantibodies especially in patients with urticarial lesions provided indirect support to the idea that IgE autoantibodies contribute to tissue damage and to certain distinct clinical features, most likely by triggering mast cell and basophil histamine release (19). Moreover, anti-BP180 IgG (46) and IgE (47) autoantibodies have been demonstrated to elicit the internalization of BP180 also through pinocytic mechanisms.

## Blister Formation Mechanisms: Update and New Theories

### Complement Cascade

The role of classical and, to a lesser extent, alternative pathways of the complement cascade in BP blister formation is fully acknowledged (48). The strength of this assumption based mainly on *in vitro* and *ex vivo* experimental models has been recently reinforced by an observational study on a large cohort of BP patients (*n* = 301) showing complement deposits in 83.1% of skin biopsies (49). These authors proved also that complement deposition was related to clinical and serological disease activity. Similarly, Chiorean et al. (50) strengthened the relevance of complement activation in BP pathogenesis by substantiating that complement activation by autoantibodies *ex vivo* as measured by the complement-fixation assay in serum correlated to disease activity. Furthermore, Kasprick et al. (51) recently assessed the impact of a mouse monoclonal IgG2 antibody inhibiting the activation of complement component 1s (C1s), a classic complement pathway-specific serine protease, on the classic complement pathway inhibition. Using cryosections of human skin incubated with serum of BP patients and a complement source leading to complement deposition along the BMZ of skin section, the authors confirmed that this monoclonal antibody was able to block the classic complement pathway activation in this model, thus providing favorable data for further studies on anti-complement monoclonal antibodies in BP patients. On the other hand, complement-dependent mechanisms may trigger independent ones in the blister formation process. Indeed, in a recent knock-out mice and pharmacological inhibition study (52), the activated fifth component of complement (C5a) along with its receptor 1 (C5aR1) appeared to be involved in the early phase of the disease, while C5a receptor 2 (C5aR2) seemed to be protective. Thus, as soon as the inflammatory process has completely developed, reactive oxygen species (ROS) and proteases are released, particularly by neutrophils and mast cells, regardless of complement involvement (45).

### Pinocytosis

In accordance with the hypothesis that anti-BP180 IgG may induce BP180 internalization from the keratinocyte cell membrane through pinocytosis (44), Tie et al. (53) showed that human keratinocytes incubated with BP IgG display dysfunctional mitochondria, increased production of ROS, and intercellular vesicle formation, leading eventually to keratinocyte detachment.

### Cytokine Profile

Unlike other skin inflammatory diseases, the key cytokines orchestrating the inflammatory cell recruitment in BP lesional skin have not yet been fully elucidated. However, a recent study (17) highlighted the central role of interleukin (IL)-17A, showing that mRNA levels of IL-17A were upregulated in perilesional skin of BP patients. Moreover, using cryosection of normal human skin and in mice, the same authors showed that inhibiting IL-17 with anti-IL-17 antibodies prevented BP180 IgG-induced blister formation. In addition, in the antibody transfer mouse BP model, IL-17A levels paralleled disease severity (17). Furthermore, the contribute of TNF-related weak inducer of apoptosis (TWEAK), a member of the TNF superfamily, and TWEAK/Fibroblast growth factor-inducible 14 (Fn14) interaction in inducing blister formation in BP has been investigated by Liu et al., who showed that TWEAK serum levels correlated inversely to BP180 expression and cellular adherence. Moreover, TWEAK activation resulted to trigger inflammation via extracellular signal–regulated kinases (ERK) and nuclear factor kappa-light-chain-enhancer of activated B cells (NF-κB) pathways (54).

### IgE, Eosinophils, and Mast Cells

Eosinophilic lesional infiltrates and peripheral eosinophilia are well-known features of BP (55–57). High levels of cytotoxic proteins stored in the secretory granules, such as eosinophil cationic protein and major basic protein, as well as a Th2 milieu associated with augmented levels of IL-4, IL-5, and IL-13, the main cytokines involved in eosinophil biology, are usually seen both in lesional skin and serum of BP patients (55, 58). In addition, eosinophils have been recognized also as a major source of IL-31, a cytokine playing a significant role in itch-related inflammation, in BP (59). The release of matrix metalloproteinase-9 (MMP-9) and the production of ROS have been found to be one of the main events depending on eosinophil involvement (56, 60). In addition, eosinophils have been hypothesized to have a strict relationship with anti-BP180 IgE autoantibodies, whose pathogenic role in BP has increasingly recognized in recent years (61, 62). Lin et al. recently proved that eosinophils are crucial for IgE-mediated blister formation, showing that binding of anti-BP180-NC16A IgE to basal keratinocytes induced eosinophil recruitment in a humanized high-affinity IgE receptor (FcεRI) mouse model of BP (63). Moreover, the interplay between FcεRI, that have been observed to be highly expressed on eosinophils in BP patients (64), and anti-BP180 IgE resulted essential in eosinophil degranulation and consequent blister formation (63). Conceivably, FcεRI plays a fundamental role also in activation of mast cell-induced inflammation in BP lesional skin (65).

### Coagulation Cascade

Eosinophils have also been demonstrated to be the main source of tissue factor (TF), an initiator of blood coagulation, in BP, representing an important link with the activation of the coagulation cascade, an ancillary mechanism contributing to blister formation. Moreover, plasma and blister fluid F1+2 prothrombin fragments (F1+2) and D-dimer levels paralleled blood and tissue eosinophilia. In turn, TF fostered the early trans-endothelial migration of eosinophils (66) and increased skin expression of adhesins and matrix metalloproteinases (67). Furthermore, blister fluid levels of eosinophilic cationic protein, a protein released during degranulation of eosinophils, positively correlated to both F1+2 and D-dimer levels (68). These findings, in conjunction with the higher expression of TF in skin biopsies of BP patients as compared to healthy controls (69, 70), outline the role of coagulation cascade in BP pathogenesis.

### Regulatory T- and B-Cell Dysfunction

The essential role of T_reg_ cells in preventing the spontaneous generation of BP autoantibodies has been recently demonstrated by Muramatsu et al. in a Stat6 (signal transducer and activator of transcription 6) gene knockout scurfy mouse model (40). The same authors identified autoantibodies to BP180 and BP230 in patients with immune dysregulation, polyendocrinopathy, enteropathy, X-linked (IPEX) syndrome and underlined an association between anti-BP180 antibody levels and an eczematous skin phenotype (40). Moreover, the absence of T_reg_ cells led to the appearance of histological subepidermal blisters in scurfy mice, with the production of pathogenic autoantibodies targeting BP230 (41). Consistent with these findings, Bieber et al. (71) demonstrated in pemphigoid mouse models that T_reg_ impair the migratory capabilities of myeloid cells by downregulating their expression of β_2_-integrin rather than their release of ROS, thus alleviating the disease severity. In addition to T_reg_, also B regulatory (B_reg_) cells have been hypothesized to play a pathogenic role in BP. In fact, Liu et al. (72) showed that B_reg_ cells from BP patients had a proinflammatory cytokine profile and were defective in suppressing CD4^+^ T cell proliferation and anti-BP180 autoantibody production, suggesting that B_regs_ play a pro-inflammatory role rather than a regulatory role in BP.

### Neutrophils and Monocytes

In addition to the well-recognized role of lymphocytes and eosinophils, it is clearly established that a cross-talk among other immune cells, notably neutrophils and monocytes, contributes in BP pathophysiology. The role of monocyte/neutrophil interaction in blister formation has been recently investigated by de Graauw et al. (73), who have highlighted in an *ex vivo* BP model that monocytes enhance the capability of neutrophils to release MMP-9, thus resulting in a more pronounced pathogenic potential and dermal-epidermal splitting. In a recent study, Fang et al. (74) provided strong evidence that BP blister fluid-derived exosomes, a subtype of extracellular vesicles involved in the transmission of inflammation signaling, recruited neutrophils through the upregulation of C-X-C Motif Chemokine Ligand 8 (CXCL8).

### Proteolytic Enzymes

Among proteolytic enzymes implied in blister formation, neutrophil elastase, and MMP-9 are the most studied. However, the role of other proteases, such as granzyme B is lately emerging. Although its pro-apoptotic functions in cytotoxic T lymphocyte-mediated are more well-known, Russo et al. have recently displayed that granzyme B is elevated in the context of dermal-epidermal junction of lesional BP skin and contribute to its disruption by cleaving BP180 (75).

## Links Between Bullous Pemphigoid Pathogenesis and Disease-Related Comorbidities

### Neurologic and Neurodegenerative Disorders

Several epidemiological studies have confirmed that patients affected by neurologic and neurodegenerative diseases, such as multiple sclerosis and Alzheimer's disease, have an increased risk of developing BP (76). Since BP180 has been described to be expressed at low levels in neuronal tissue (77), it has been argued that neurodegeneration or neuroinflammation may lead to loss of self-tolerance toward BP180 and consequent BP development. Although a certain degree of cross-reactivity between BP180 and/or BP230 brain and skin isoforms exist (78), a recent study (79) evidenced that in Alzheimer's disease and multiple sclerosis patients the autoantibodies against BP180 targeted epitopes different than those targeted in patients with BP and did not bind natively folded BP180, thus suggesting their low potential to induce BP. Future studies could investigate the role of epitope spreading phenomenon in the induction of BP (80).

### Vitamin D Deficiency

A higher prevalence of vitamin D deficiency and lower 25-hydroxyvitamin D levels were found in BP patients as compared to controls by our group (81). An extended study additionally showed that 25-hydroxyvitamin D levels inversely correlated to disease severity (82). Indeed, vitamin D has been shown to have an immune regulatory function and hypovitaminosis D has been correlated to an increased risk of developing autoimmune disorders (83). Moreover, vitamin D3 has been revealed to elicit down-regulation of the BP230 gene through post-transcriptional mechanisms independently by active protein synthesis (84), while non-toxic doses of calcitriol, the hormonally active vitamin D metabolite, reduced the release of the proinflammatory cytokines IL-6 and IL-8 induced by purified human BP IgG from HaCaT cells (85). A similarly beneficial effect of calcitriol has been recently reported also in mice with experimental EBA (86).

### Thrombosis

The risk of thrombosis and/or thromboembolism is augmented in BP patients (87), as witnessed by a recent multicenter cohort study showing that BP patients have a 4-fold increased risk of developing venous thromboembolism compared to the general population (88). Indeed, several studies revealed that the inflammatory response in patients with BP activated the coagulation cascade, which paralleled disease activity (66, 70, 89, 90). In particular, D-dimer and F1+2, two coagulation activation markers, have been observed to be increased in blister fluid, lesional skin and plasma (90), with F1+2 correlating to the anti-BP180 levels (89). Moreover, during remission phases, blood concentration of coagulation activation markers reverted to normal (70). Therefore, extrinsic blood coagulation pathway activation may be regarded as a contributory mechanism leading to local inflammation and blister formation in BP (79, 80, 82). Fibrinolysis resulted also inhibited in BP, chiefly due to increased plasminogen activator inhibitor type 1 (PAI-1) activity and plasma levels (91). Interestingly, the recent observation that serum levels of soluble P-selectin, a marker of platelet activation, were significantly higher in BP patients than controls led to hypothesize a role of platelets in disease pathogenesis (92).

## Conclusion

As highlighted in this review, the pathogenesis of BP is complex, and evidence is growing that new ancillary pathomechanisms flanking the traditional ones—e.g., IgG1-mediated complement activation—may play a role in the events leading to subepithelial blister formation.

## Author Contributions

GG and AM designed and wrote the initial draft of the manuscript. GD, EC, EB, and MC reviewed the paper and provided critical intellectual input. All the authors approved the final version of the manuscript.

### Conflict of Interest Statement

The authors declare that the research was conducted in the absence of any commercial or financial relationships that could be construed as a potential conflict of interest.
